# Enhanced CYP2C19-mediated drug-drug interaction risk with escitalopram in geriatric populations

**DOI:** 10.3389/fphar.2025.1711196

**Published:** 2026-01-05

**Authors:** Seonyoung Byoun, Dong-Gyu Heo, Minsoo Lee, Ryunghwa Lee, Yuanyuan Li, Ju-Yeun Lee, Eunjin Hong, Wooin Lee

**Affiliations:** 1 College of Pharmacy and Research Institute of Pharmaceutical Sciences, Seoul National University, Seoul, Republic of Korea; 2 College of Pharmacy, Dongguk University-Seoul, Goyang-si, Republic of Korea

**Keywords:** escitalopram, CYP2C19, geriatric population, drug-drug interactions, PBPK modeling

## Abstract

**Introduction:**

Escitalopram (S-CIT) is commonly prescribed for depression and anxiety in older patients. Previous research has reported that the effect of CYP2C19 polymorphism on S-CIT pharmacokinetics is more pronounced in older adults than in young adults. The current study investigated whether older adults taking S-CIT face a greater risk for CYP2C19-mediated drug-drug interaction (DDI) than young adults.

**Methods:**

Using a physiologically-based pharmacokinetic (PBPK) model, we quantitatively compared the risk of CYP2C19-mediated DDIs in older adults taking S-CIT with any of four CYP2C19 inhibitors (omeprazole, esomeprazole, fluconazole, and fluoxetine). These CYP2C19 inhibitors were selected based on their prevalence of co-administration with S-CIT, as determined by a retrospective analysis of the 2019 Korean National Health Insurance Service senior cohort database.

**Results and Discussion:**

Our PBPK modeling-based simulations predicted that the extent of DDI incurred by S-CIT would be greater in older adults than in young adults and vary significantly by CYP2C19 phenotypes (extensive, intermediate, and poor metabolizers). Based on the prediction results, we propose CYP2C19 phenotype-guided S-CIT dosing strategies for older adults. Implementing the proposed dosing recommendation may reduce the incidence of potentially inappropriate use of medication and adverse events in older adults prescribed S-CIT.

## Graphical Abstract

**Figure:**
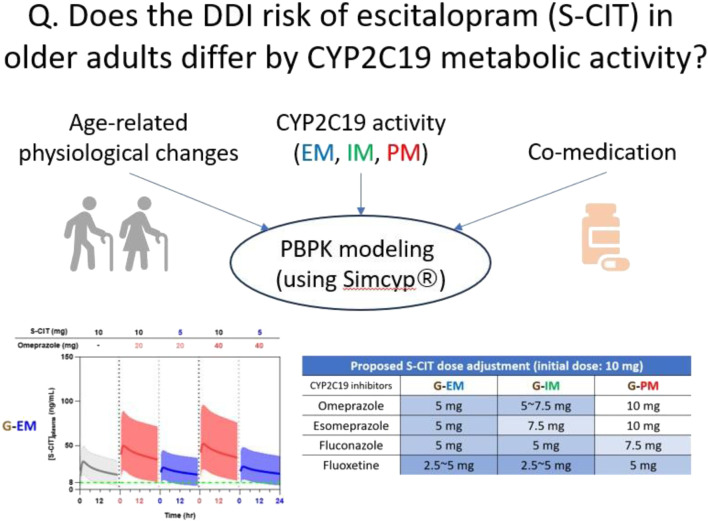

## 1 Introduction

Late-life depression (LLD) is a common mental disorder among older adults, with a 2024 meta-analysis estimating its global prevalence at 19.2% (Jalali et al., 2024). The burden of LLD is projected to increase, considering global demographic shifts; the proportion of the world’s population aged 65 or older is expected to rise from approximately 10% in 2024 to 16% by 2050 (UN, 2024). In addition to diminishing the quality of life and independence, LLD is also associated with increased mortality (Fiske et al., 2009). Notably, 54%–87% of older adults who died by suicide had a prior diagnosis of major depressive disorder, underscoring the life-saving potential of early detection and effective treatment of LLD (Conwell and Thompson, 2008).

Escitalopram (S-CIT), a selective serotonin reuptake inhibitor (SSRI), is a first-line therapy for LLD in older patients. S-CIT exhibits an absolute oral bioavailability (F) of approximately 80% and moderate plasma protein binding of about 56% (LEXAPRO package insert). In healthy volunteers, multiple 10 mg daily doses (QD) result in a mean C_max_ of 20.63 ng/mL and a mean t_max_ of 3.9 h (Rao, 2007). Hepatic metabolism of S-CIT is mediated by CYP2C19, CYP3A4, and CYP2D6 (Rao, 2007). S-CIT exposure may increase when co-administered with medications that inhibit CYP2C19, such as certain proton pump inhibitors (PPIs) and azole antifungals. Clinical and observational evidence suggest that such interactions can elevate systemic S-CIT concentrations and thereby increase the risk of dose-dependent QTc interval prolongation, a particular concern in older adults. Rare but clinically significant adverse events, including serotonin toxicity and hyponatremia, have also been reported (Personne et al., 1997; Roncero et al., 2013; Rawal et al., 2017; Assimon et al., 2022; Wu et al., 2023). These safety concerns are reflected in product labeling, with recommendations for caution and monitoring (e.g., ECG monitoring and assessment of electrolytes), as well as dose adjustments when strong CYP2C19 inhibitors are co-prescribed (LEXAPRO package insert).

Age-related physiological changes, including reduced hepatic and renal function, could lead to elevated plasma concentrations of S-CIT in older patients, increasing their susceptibility to adverse drug reactions (Rao, 2007). Older adults face elevated systemic exposure of S-CIT and a higher risk of dose-dependent QTc interval prolongation (Rao, 2007; Beyer and Johnson, 2018). Consequently, the U.S. Food and Drug Administration (FDA) recommends a maximum 10 mg QD for older patients, half the dose approved for younger adults (LEXAPRO package insert). However, substantial interindividual pharmacokinetic (PK) and pharmacodynamic (PD) variability have been observed among older patients taking the reduced S-CIT dose of 10 mg QD. For example, a clinical trial involving patients aged 65–93 years demonstrated that S-CIT 10 mg QD produced highly variable therapeutic responses, with 9.8% of patients discontinuing treatment due to intolerable side effects (Kasper et al., 2005). These results suggest that a simple maximum dose-reduction approach may not be sufficient for managing S-CIT therapy in older patients.

In addition to age-related physiological changes, CYP2C19 polymorphism and CYP2C19-mediated drug–drug interactions (DDIs) may further complicate S-CIT therapy in older patients. A recent study demonstrated that CYP2C19 phenotypes have a more pronounced impact on S-CIT exposure in older adults than in younger individuals, based on both clinical data and physiologically based PK (PBPK) modeling (Jang et al., 2025). For CYP2C19, the relationship between genotypes and phenotypes is established for frequently observed genetic variants, but less so for other genotypes. Generally, individuals carrying the *1/*1 genotype were classified as extensive metabolizers (EM), those with *1/*2 or *1/*3 as intermediate metabolizers (IM), and those with *2/*2, *2/*3, or *3/*3 as poor metabolizers (PM). For other CYP2C19 genotypes, however, a standardized, consensus-based activity scoring system has yet to be established.

Given the high prevalence of polypathology and polypharmacy in older adults, the risk of CYP2C19-mediated DDIs is likely to be elevated in this population (Lattanzio et al., 2012). The extent of DDIs can vary depending on individual metabolic capacity, which is affected by age and CYP2C19 phenotype. This introduces further complexity to the safe and effective use of S-CIT in older patients. Yet, the DDI risk associated with S-CIT in older adults has not been quantitatively characterized. This knowledge gap is critical, as DDIs may further elevate S-CIT exposure in older patients predisposed to higher drug exposure due to aging. Therefore, a comprehensive evaluation of CYP2C19-mediated DDI risk in older patients, stratified by CYP2C19 phenotype, is necessary to better understand the variability in drug exposure and to devise individualized dosing strategies for S-CIT.

In the current study, we aimed to: i. quantitatively compare the risk of CYP2C19-mediated DDIs involving S-CIT as a victim drug and four frequently co-prescribed CYP2C19 inhibitors in young and older adults; and ii. evaluate the extent of DDIs in older adults stratified by CYP2C19 phenotype, with the goal of proposing phenotype-specific dosing recommendations. This study may provide a foundation for developing individualized geriatric dosing strategies for S-CIT, tailored to both concomitant medications and CYP2C19 phenotype.

## 2 Materials and methods

### 2.1 Real-world prevalence of concomitant CYP2C19 inhibitor use in older patients receiving S-CIT in Korea

We conducted a cross-sectional study using the Korean National Health Insurance Service (NHIS) Senior cohort database (2003–2019) to examine the real-world prevalence of co-medications involving S-CIT and CYP2C19 inhibitors in older adults. The NHIS cohort comprised an 8% random sample of Korean adults aged 60–80 years. The cohort initially included 511,953 individuals, and was annually supplemented with 8% of newly eligible 60-year-olds from 2009 to 2019, adding 545,831 subjects by 2019. The database provides detailed information on demographics, healthcare utilization, and prescription claims. Supplementary Table S1 summarizes the demographic and clinical characteristics of S-CIT users and patients receiving co-administered CYP2C19 inhibitors. In all exposure categories, the majority of patients were in their 70 s, with a consistently higher proportion of female patients. Mean ages ranged from 70.1 to 73.5 years. This study protocol was reviewed and approved as exempt from review by the Institutional Review Board of Seoul National University (IRB No. E2503/004-001).

Among 895,954 geriatric adults who received at least one prescription in 2019, we identified 31,829 individuals who had been prescribed S-CIT at least once, encompassing a total of 203,023 S-CIT prescriptions. To quantify co-medication prevalence, 12 oral CYP2C19 inhibitors were selected based on the three sources - the Micromedex Drug Interaction database, the U.S. FDA Drug Development and Drug Interaction Guidance, and the Indiana University Flockhart Table™ - and their availability as oral medications. The selected drugs included omeprazole, esomeprazole, lansoprazole, cimetidine, voriconazole, fluconazole, fluvoxamine, fluoxetine, ticlopidine, modafinil, armodafinil, and isoniazid (Supplementary Table S2). For the selected medications, we used two complementary prevalence measures: (a) patient-level prevalence, defined as the proportion of S-CIT users who were exposed to at least 1 day of overlapping use with a CYP2C19 inhibitor during the treatment episode; and (b) person-day prevalence, defined as the proportion of S-CIT person-days that overlapped with CYP2C19 inhibitor use. In addition, we compiled the frequencies of S-CIT doses prescribed to older adults.

All analyses were performed using SAS version 9.4 (SAS Institute Inc., Cary, NC, USA). For PBPK modeling-based DDI simulations, a final set of four CYP2C19 inhibitors - omeprazole, esomeprazole, fluconazole, and fluoxetine - was selected (Table 1). The initial list of candidates for DDI prediction included the six CYP2C19 inhibitors that are most frequently co-prescribed with S-CIT: esomeprazole, cimetidine, lansoprazole, fluconazole, omeprazole, and fluoxetine. However, cimetidine and lansoprazole were ultimately excluded from the DDI prediction. Cimetidine was excluded due to its predicted lack of significant CYP2C19 inhibition *in vivo*, based on its low [I]/Ki (Furuta et al., 2001). Lansoprazole was excluded based on clinical data showing no meaningful CYP2C19-mediated DDIs when co-administered with CYP2C19 substrates such as diazepam and phenytoin (Goodman et al., 2011). While lansoprazole exhibits *in vitro* CYP2C19 inhibitory potential, it lacks the mechanism-based inhibition observed with omeprazole, which explains why omeprazole causes clinically significant interactions whereas lansoprazole does not (Ogilvie et al., 2011).

**TABLE 1:** Prevalence of four selected CYP2C19 inhibitor use in Korean older cohorts: A patient-level and person-day analysis in the total population and S-CIT users.

CYP2C19 inhibitors	Total population (S-CIT users and non-users) (N = 895,954, Patient-days = 254,762,256)		S-CIT users (N = 31,829, Patient-days = 5,531,604)			
	Patient-level prevalence (%)	Person-day prevalence (%)	Patient-level prevalence (%)	Person-day prevalence (%)	Duration of co-administration (days)	
					Mean ± SD	Median (IQR)
Esomeprazole	22.9	5.1	19.9	7.4	64.5 ± 88.4	27 (9–75)
Fluconazole	6.1	0.4	3.8	0.4	16.1 ± 20.8	10 (5–20)
Omeprazole	3.3	0.7	2.9	0.8	49.7 ± 73.4	18 (8–61)
Fluoxetine	0.4	0.2	1.4	0.6	73.7 ± 111.4	15 (6–87)
Sub-total (%)	32.7	6.4	28.0	9.2	-	-

### 2.2 PBPK model development

The PBPK models were implemented using the Simcyp PBPK Simulator (version 23; Certara, Sheffield, UK). We utilized the Simcyp Healthy Volunteer (18–65 years) and Geriatric North European Caucasian (65–98 years) populations for the PK simulations of young and older adults, respectively. For older adults, we created three sub-populations composed exclusively of each CYP2C19 phenotype: extensive metabolizers (EM), intermediate metabolizers (IM), and poor metabolizers (PM). The proportion of females was set to 0.67 based on a cross-sectional study using the Korean NHIS Senior cohort database. For non-stratified populations (young or older adults), simulations were conducted with a total population of 500, comprising 10 trials of 50 subjects each. For individual CYP2C19 phenotype sub-populations of older adults, simulations were conducted with a total population of 100, comprising 10 trials of 10 subjects each.

Key drug-dependent parameters of S-CIT and omeprazole were obtained from the literature. The S-CIT parameter set was mainly from the previous study (Jang et al., 2025) (Supplementary Table S3). Intrinsic metabolic clearance of S-CIT by each CYP isoform was estimated using the retrograde calculator in Simcyp, based on hepatic clearance and the fraction metabolized (f_m_) by each CYP isoform. The parameter set for omeprazole available from the Simcyp library incorporated only mechanism-based inhibition of CYP2C19. For our current analysis, we developed separate parameter sets for R- and S-omeprazole to incorporate both competitive and mechanism-based inhibition of CYP2C19 in a stereoselective manner (Wu et al., 2014) (Supplementary Table S4). The parameter set for the S-enantiomer was then used to simulate the PK profiles under DDI conditions involving esomeprazole. For fluconazole and fluoxetine, we used the compound parameter sets provided in the Simcyp library.

### 2.3 PBPK model validation

#### 2.3.1 PBPK model validation: PK simulations for S-CIT

To validate the developed PBPK model of S-CIT, eleven clinical datasets (seven single-dose studies and four multiple-dose studies) were used. In addition to the clinical datasets used in the previous study (Jang et al., 2025), four additional datasets from Korean subjects receiving a single administration of 5–30 mg S-CIT were included (Kim et al., 2017). The clinical datasets were from the studies in young adults, except for one focused on older adults (Gutierrez and Mengel, 2002). For simulations, drug doses, administration schedules, ages, and sex distributions were matched to the design of the corresponding clinical studies as summarized in Supplementary Table S5 (Gutierrez and Mengel, 2002; Malling et al., 2005; Søgaard et al., 2005; Muñoz et al., 2015; Kim et al., 2017).

The ratios of the mean observed values to the mean predicted values were calculated for the maximal concentration (C_max_) and the area under the plasma concentration-time curve (AUC). Model performance was considered acceptable if the simulated values fell within a two-fold range of the observed values (Wang, 2010).

#### 2.3.2 PBPK model validation: DDI simulations

The PBPK model of S-CIT was further assessed using the clinical DDI data with omeprazole to determine its suitability for predicting PK changes under DDI conditions. The clinical dataset (Malling et al., 2005) involved a single dose of 20 mg S-CIT administered to healthy volunteers receiving omeprazole (30 mg QD for 5 days). For simulations, the dose and schedule of drugs were matched to the design of the reported clinical DDI study (Malling et al., 2005). DDI magnitude was quantified by calculating the arithmetic mean values of S-CIT AUC and C_max_ ratios under control and DDI conditions. DDI prediction was considered acceptable when the simulated ratios fell within the 0.80–1.25 range of the observed clinical values (ICH, 2024).

### 2.4 PBPK model application

#### 2.4.1 S-CIT as a victim drug: simulating DDI with four CYP2C19 inhibitors in young and older adults

The validated PBPK models were applied to predict the extent of DDI between S-CIT and four CYP2C19 inhibitors (omeprazole, esomeprazole, fluconazole, and fluoxetine) in both older and young adult populations. Simulations were performed for non-stratified young and older adult populations, as well as for older adult sub-populations stratified by CYP2C19 phenotype: G-EM, G-IM, and G-PM. The dosing regimens for each CYP2C19 inhibitor were selected based on their respective package inserts: omeprazole, 20 or 40 mg QD; esomeprazole, 10 or 20 mg QD; fluconazole, 100 or 200 mg QD; fluoxetine, 20 or 40 mg QD. S-CIT was simulated at 10 mg QD (the maximum recommended dose for older patients by the US FDA). S-CIT and each CYP2C19 inhibitor were co-administered once daily from Day 1 at the specified doses. Steady-state simulations were performed following 30 days of CYP2C19 inhibitor administration, except for fluoxetine, which was given for 60 days to account for its prolonged time to reach steady-state.

Comparisons of simulated C_max_ and AUC ratios between non-stratified young and older adult populations were conducted using Welch’s t-test (assuming unequal variances) (Figure 3; Supplementary Figure S2). For the older adult sub-populations stratified by CYP2C19 phenotype, pairwise comparisons of simulated C_max_ and AUC ratios between G-EM and G-IM were performed using Welch’s t-test for omeprazole and esomeprazole. For fluconazole and fluoxetine, differences among G-EM, G-IM, and G-PM were assessed using one-way ANOVA followed by Tukey’s multiple comparisons test (Figure 4). The statistical analysis for comparing simulated DDI magnitudes was chosen based on the number of phenotype groups included in each comparison. For omeprazole and esomeprazole, comparisons involved only two phenotype groups (G-EM vs. G-IM); therefore, Welch’s t-test was used to compare simulated C_max_ and AUC ratios between the two groups, given its robustness against unequal variances. For fluconazole and fluoxetine, all three phenotype groups (G-EM, G-IM, and G-PM) were included. We used a one-way ANOVA to assess overall differences, followed by Tukey’s multiple comparisons test for pairwise comparisons.

#### 2.4.2 Potential dose adjustment of S-CIT for older adults taking four CYP2C19 inhibitors

PBPK modeling-based simulations for S-CIT as a victim drug were performed to guide S-CIT dose adjustments when co-administered with any of four CYP2C19 inhibitors. Simulations were performed to determine the S-CIT dose adjustment (reduction by 25%, 50%, or 75% from the maximum recommended dose of 10 mg in older adults; i.e., 7.5, 5, or 2.5 mg) that would result in equivalent S-CIT exposure (steady-state S-CIT AUC values with fold differences within 0.80–1.25) of the control condition (S-CIT monotherapy).

## 3 Results

### 3.1 Real-world prevalence of concomitant CYP2C19 inhibitor use in older patients receiving S-CIT in Korea

Among Korean older adults with at least one prescription in 2019 (including both S-CIT users and non-users), 407,410 patients received at least one of the predefined CYP2C19 inhibitors (Supplementary Table S2). Esomeprazole, cimetidine, and fluconazole were the most prescribed agents. Of the 31,829 S-CIT users, 38.6% were found to have a concomitant use with a CYP2C19 inhibitor. In terms of patient-level prevalence, the co-administered CYP2C19 inhibitors were as follows: esomeprazole (19.9%), fluconazole (3.8%), omeprazole (2.9%), and fluoxetine (1.4%) (Table 1). Based on person-day prevalence, esomeprazole accounted for the highest proportion of overlapping treatment days (7.4%, 408,581 days), with a mean duration of co-administration being 64.5 days. This was followed by omeprazole (0.8%, 46,373 days), fluoxetine (0.6%, 33,823 days), and fluconazole (0.4%, 19,244 days), with mean durations of 49.7, 73.7, and 16.1 days, respectively (Table 1). Rarely used agents, including modafinil, armodafinil, and voriconazole, contributed minimally to total patient-days (Supplementary Table S2).

**FIGURE 1:**
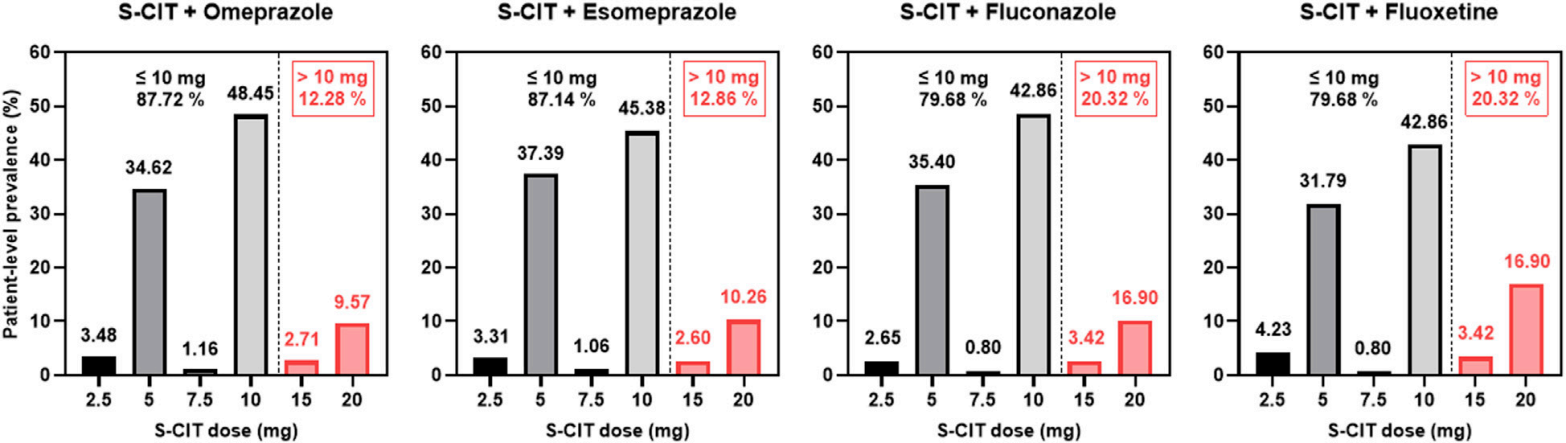
Real-world distribution of S-CIT doses in Korean older adults taking S-CIT and its co-administration with CYP2C19 Inhibitors.

For Korean older patients taking S-CIT and any of the four CYP2C19 inhibitors (esomeprazole, omeprazole, fluconazole, or fluoxetine), the real-world distribution data of S-CIT doses were compiled (Figure 1). Despite the US FDA’s recommendation for a maximum daily S-CIT dose of 10 mg QD in the geriatric population, the analysis indicated that over 10% of Korean older patients received S-CIT daily doses exceeding 10 mg. Among the older patients who received both S-CIT and any of the four CYP2C19 inhibitors, the patient-level prevalence for S-CIT daily doses exceeding 10 mg ranged from 12.28% to 20.32% (Figure 1).

### 3.2 PBPK model validation

The PBPK model of S-CIT was validated using 11 clinical datasets. In the previous study (Jang et al., 2025), validation was performed with seven clinical datasets mostly from the Caucasian population (Gutierrez and Mengel, 2002; Malling et al., 2005; Søgaard et al., 2005; Muñoz et al., 2015; Kim et al., 2017). Overall, the simulated PK profiles agreed well with the observed data, which fell within the 5%–95% range of the simulation results (Supplementary Figure S1). Across all datasets, the predicted C_max_ values were 0.86–1.60 times the observed C_max_ values, and the predicted AUC values ranged from 0.72 to 1.26 times the observed AUC values (Supplementary Table S5).

The PBPK model adequately captured the PK profiles reported in Korean subjects receiving S-CIT doses ranging from 5 to 30 mg (Kim et al., 2017). These results suggested that the predictive performance of the current PBPK model of S-CIT was comparable between Caucasian and Korean subjects. The analyzed dataset included one study conducted in older adults (65–80 years) (Gutierrez and Mengel, 2002). The current PBPK model of S-CIT well captured the observed data with simulated-to-observed ratios of 1.04 and 0.97 for C_max_ and AUC, respectively. The model predicted that mean C_max_ and AUC values would be higher in older adults than in young adults, with a 1.59-fold increase for C_max_ (31.60 vs. 19.80 ng/mL) and 1.85-fold increase for AUC (530.70 vs. 287.00 h∙ng/mL) (Supplementary Table S5). These predictions closely matched the reported data (Gutierrez and Mengel, 2002).

**FIGURE 2:**
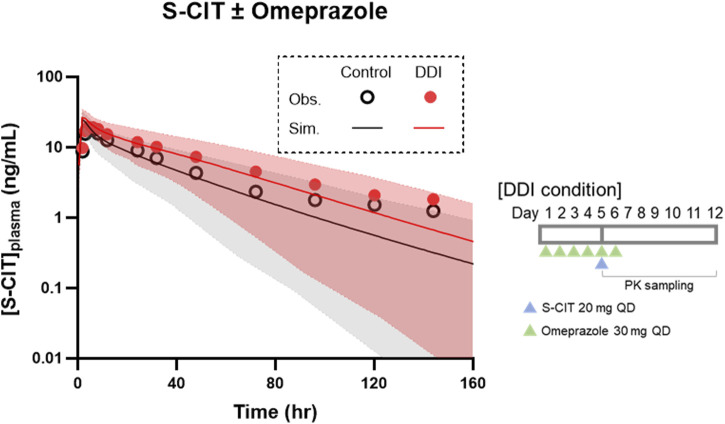
DDI predictive performance of the PBPK model of S-CIT in the DDI condition with omeprazole. Simulated PK profiles are shown as lines and shadows. Observed clinical data are shown in symbols: control conditions (open circles) and DDI conditions (closed circles).

To apply the PBPK model of S-CIT for the characterization of DDIs, it is essential to verify its suitability for assessing victim DDI liability. The robustness of the model was assessed by comparing the magnitude of the simulated DDI between S-CIT and omeprazole with that observed in clinical trials conducted in a young adult population (Malling et al., 2005). The PBPK models accurately recapitulated the observed DDI magnitude. As summarized in Table 2, the ratios of C_max_ and AUC under DDI conditions predicted by simulations closely matched the observed data: the simulated-to-observed ratios of 0.99 and 1.01, respectively. The observed and simulated PK profiles of S-CIT under DDI conditions were generally consistent, except beyond 100 h post-dose, where a slight underprediction was noted (Figure 2).

**TABLE 2:** Predictive performance of the PBPK models of S-CIT and omeprazole in capturing the ratios (DDI/control) in PK parameters (C_max_ and ${\text{AUC}}_{\left(0-\infty \right)}$ ) under DDI conditions.

PK parameters	Condition	Observed (Malling et al., 2005)	Simulated (SD)	Ratio (Simulated/Observed)
C_max_ (ng/mL)	Control	19.59	25.19 (4.68)	1.29
	DDI	21.31	27.08 (4.79)	1.27
	Ratio (DDI/control)	1.09	1.08	0.99
${\text{AUC}}_{\left(0-\infty \right)}$ (h^⋅^ng/mL)	Control	628.34	578.54 (265.22)	0.92
	DDI	950.14	880.28 (369.66)	0.93
	Ratio (DDI/control)	1.51	1.52	1.01

### 3.3 DDI prediction

#### 3.3.1 DDI prediction for S-CIT (10 mg QD) and four CYP2C19 inhibitors

**FIGURE 3:**
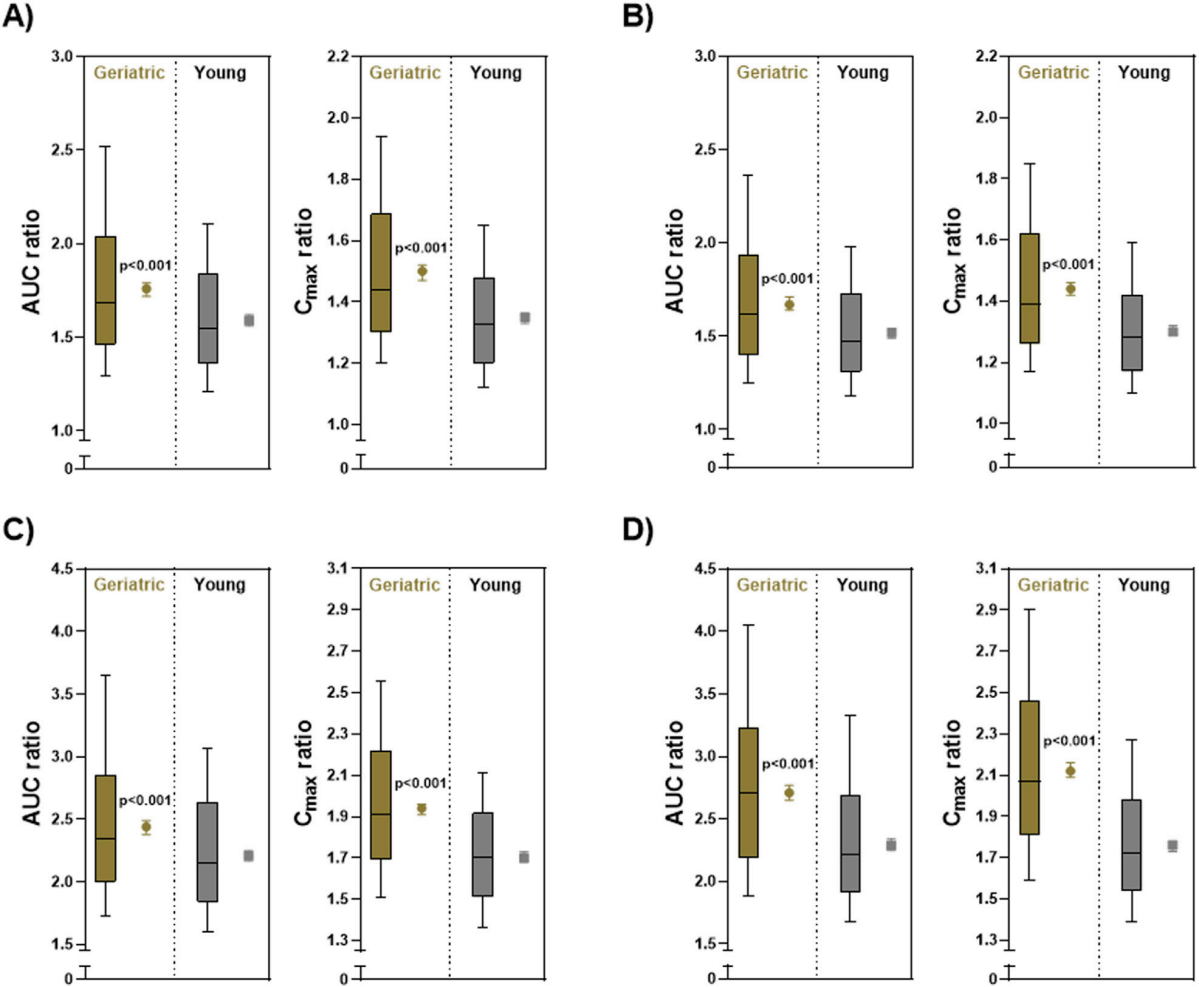
Simulated C_max_ and AUC ratios (at steady-state) in non-stratified young and older adults (N = 500) for S-CIT under DDI conditions. Distributions are shown as a box-and-whisker plot where the box represents the interquartile range, and the whiskers extend to 10%–90%. Geometric means are shown as a dot plot with 90% confidence intervals. **(A)** Omeprazole 20 mg QD, **(B)** Esomeprazole 10 mg QD, **(C)** Fluconazole 100 mg QD, and **(D)** Fluoxetine 20 mg QD. *P* values were determined using a Welch’s t-test (assuming unequal variances).

We conducted simulations of DDI conditions involving S-CIT and four CYP2C19 inhibitors in non-stratified young and older adults. The systemic exposure of S-CIT was substantially elevated when co-administered with any of the four CYP2C19 inhibitors in both young and older adults (Figure 3). Similar trends were observed when the doses of CYP2C19 inhibitors were increased by two-fold (Supplementary Figure S2). For all CYP2C19 inhibitors evaluated, the distribution of AUC and C_max_ ratios appeared wider in older adults than in young adults, and the geometric means of AUC and C_max_ ratios were significantly higher in older adults than in young adults (p < 0.0001, Figure 3).

Among the CYP2C19 inhibitors tested, fluoxetine (20 mg QD) exhibited the greatest DDI magnitude, with the predicted AUC ratios of 2.71 (90% confidence interval (CI): 2.65, 2.77) in older adults and 2.29 (90% CI: 2.25, 2.34) in young adults (Figure 3D). For the other CYP2C19 inhibitors, the predicted AUC ratios in older and young adult populations were as follows: 2.44 (90% CI: 2.38, 2.49) and 2.21 (90% CI: 2.17, 2.25) for fluconazole 100 mg; 1.67 (90% CI: 1.64, 1.71) and 1.52 (90% CI: 1.49, 1.54) for esomeprazole 10 mg; and 1.76 (90% CI: 1.72, 1.79) and 1.59 (90% CI: 1.56, 1.62) for omeprazole 20 mg (Figures 3A–C).

**FIGURE 4:**
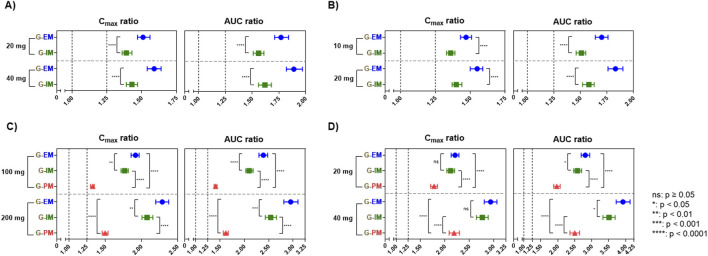
Geometric means (with 90% confidence intervals) of simulated C_max_ and AUC ratios (at steady-state) in older adult sub-populations of differing CYP2C19 phenotypes: CYP2C19 extensive metabolizer (EM), intermediate metabolizer (IM), and poor metabolizer (PM) (N = 100) for S-CIT under DDI conditions with **(A)** Omeprazole 20 or 40 mg QD, **(B)** Esomeprazole 10 or 20 mg QD, **(C)** Fluconazole 100 or 200 mg QD, and **(D)** Fluoxetine 20 or 40 mg QD. Differences between groups were determined by one-way ANOVA, followed by a *post hoc* test (Tukey’s multiple comparisons test). Statistical significance is indicated as follows: ****, *p* < 0.0001, ***, *p* < 0.001, **, *p* < 0.01, *, *p* < 0.05, ns, not significant.

We further evaluated the DDI magnitude of S-CIT in older adults stratified by CYP2C19 phenotype: G-EM, G-IM, and G-PM (Figure 4). The predicted C_max_ and AUC ratios were highest in G-EM, followed by G-IM and G-PM, consistent with the expected gradient of CYP2C19 metabolic activity across phenotypes. For omeprazole and esomeprazole (inhibitory effects on CYP2C19 only), the predicted C_max_ and AUC ratios were significantly higher in G-EM than in G-IM (p < 0.0001, Figures 4A,B). There was no DDI in G-PM, as expected from the absence of CYP2C19 activity in this phenotype. For fluconazole and fluoxetine (inhibitory effects on both CYP2C19 and CYP3A4), the magnitude of DDI was predicted to be in the following order: G-EM, G-IM, and G-PM (Figures 4C,D). Among the four CYP2C19 inhibitors, fluoxetine (40 mg QD) displayed the greatest differences in the predicted AUC ratios among CYP2C19 phenotypes: 3.92 (90% CI: 3.72, 4.13), 3.51 (90% CI: 3.33, 3.69), and 2.50 (90% CI: 2.36, 2.64) in G-EM, G-IM, and G-PM, respectively (Figure 4D).

#### 3.3.2 Simulations with adjusted S-CIT doses for older adults under DDI conditions with CYP2C19 inhibitors

**FIGURE 5:**
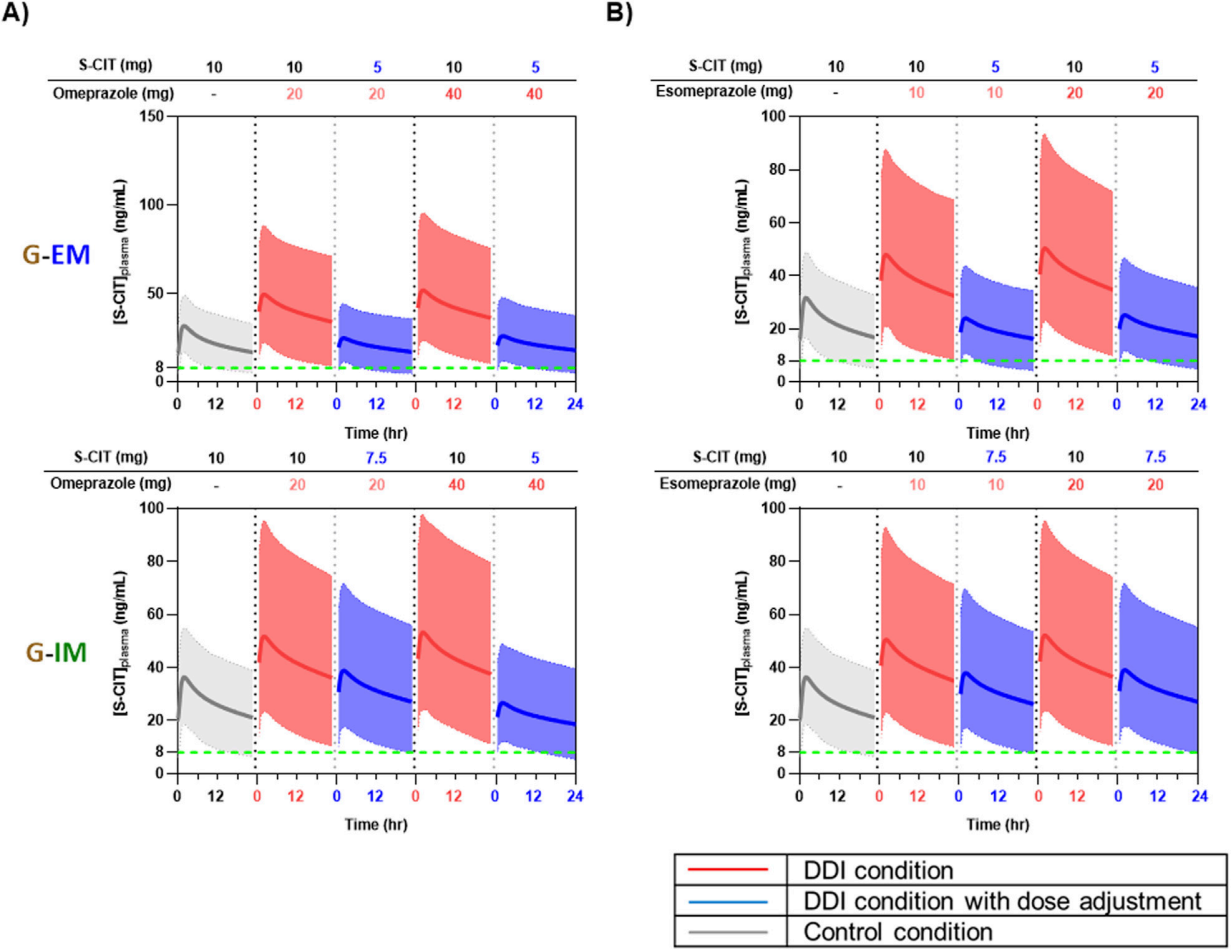
Predicted plasma concentration-time profiles of S-CIT under the control condition (10 mg S-CIT monotherapy) and the DDI condition (S-CIT with omeprazole or esomeprazole), with adjusted doses of S-CIT in CYP2C19 EM and IM sub-populations of older adults. **(A)** Omeprazole **(B)** Esomeprazole.

**FIGURE 6:**
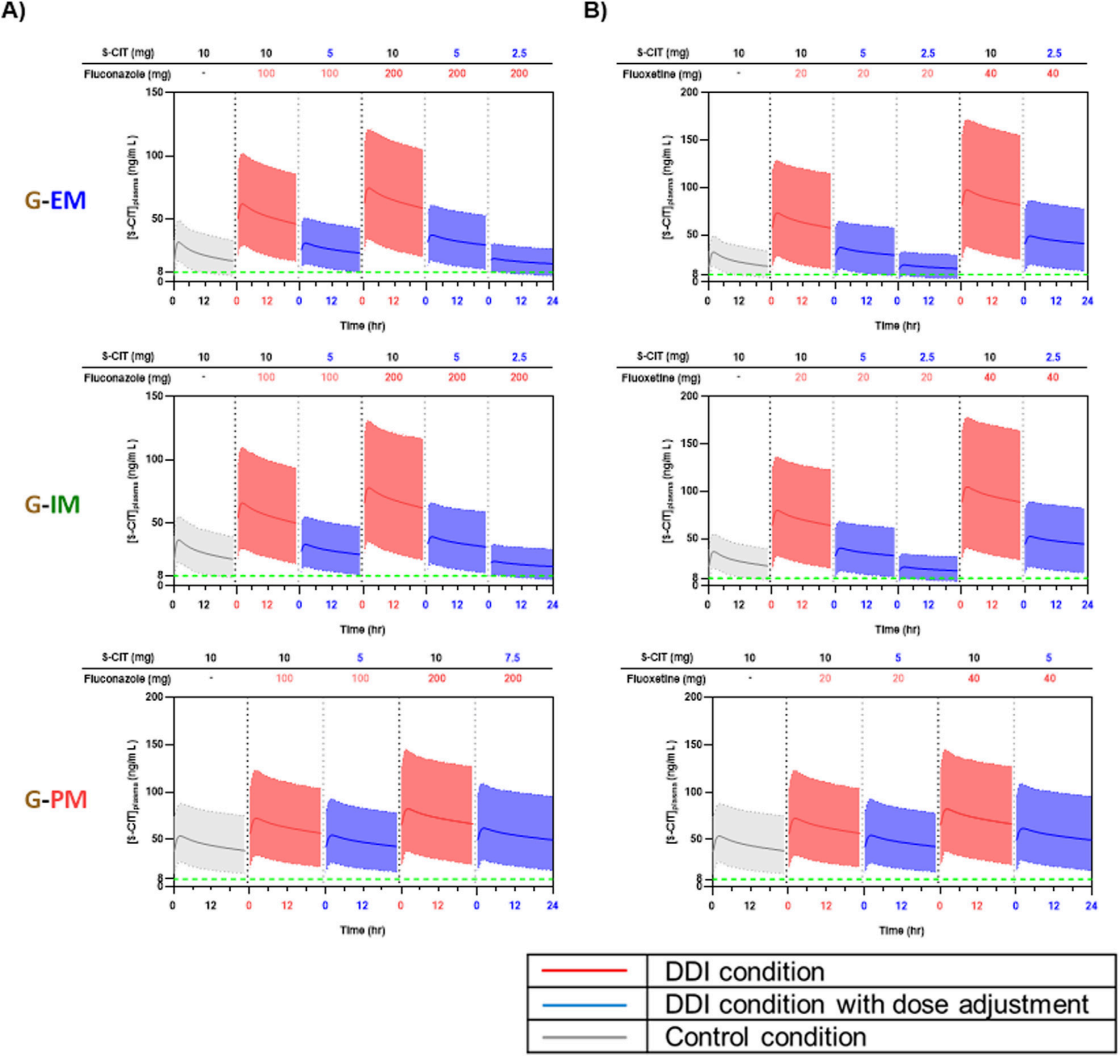
Predicted plasma concentration-time profiles of S-CIT under the control condition (10 mg S-CIT monotherapy) and in DDI condition (S-CIT with fluconazole and fluoxetine), with adjusted doses of S-CIT in CYP2C19 EM, IM, and PM sub-populations of older adults. **(A)** Fluconazole **(B)** Fluoxetine.

Figures 5, 6 show the predicted steady-state plasma PK profiles of S-CIT in G-EM and G-IM when co-administered with the CYP2C19 inhibitors. Simulations were performed to find incremental S-CIT doses that achieve equivalent S-CIT exposure in older adults under DDI conditions. The predicted C_max_ and AUC values (relative to S-CIT monotherapy), following S-CIT dose adjustment under DDI conditions with the respective CYP2C19 inhibitors, are summarized in Table 3. For co-administration with omeprazole 20 mg, adjusted S-CIT doses of 5 and 7.5 mg (reduction by 50% and 25%) led to equivalent S-CIT exposure for G-EM and G-IM, respectively (Figure 5A). When co-administered with omeprazole 40 mg, an adjusted S-CIT dose of 5 mg (reduction by 50%) resulted in equivalent S-CIT exposure for both G-EM and G-IM (Figure 5A). In the case of esomeprazole at both 10 mg and 20 mg doses, adjusted S-CIT doses of 5 mg (reduction by 50%) and 7.5 mg (reduction by 25%) were recommended for G-EM and G-IM, respectively (Figure 5B).

**TABLE 3:** Predicted C_max_ and AUC values (at steady-state) with adjusted S-CIT doses (25% increments) under DDI conditions involving the four CYP2C19 inhibitors in older patients of differing CYP2C19 phenotypes (G-EM, G-IM, and G-PM). The adjusted S-CIT doses achieving equivalent AUC values to control conditions are marked in bold.

CYP2C19 inhibitor and dose (mg)		CYP2C19 phenotype	S-CIT adjusted dose (mg, % reduction)	Relative exposure with an adjusted S-CIT dose (compared to control condition)	
				AUC (%)	Cmax (%)
Omeprazole	20	G-EM	**5 (50% ↓)**	88.69	75.69
		G-IM	**7.5 (25% ↓)**	116.64	104.34
			5 (50% ↓)	77.76	69.54
	40	G-EM	**5 (50% ↓)**	94.58	79.49
		G-IM		80.84	71.68
Esomeprazole	10	G-EM	**5 (50% ↓)**	85.04	73.30
		G-IM	**7.5 (25% ↓)**	113.12	101.85
			5 (50% ↓)	75.41	67.92
	20	G-EM	**5 (50% ↓)**	91.25	77.27
		G-IM	**7.5 (25% ↓)**	118.18	105.35
			5 (50% ↓)	78.79	70.23
Fluconazole	100	G-EM	**5 (50% ↓)**	119.47	95.87
		G-IM		105.04	88.69
		G-PM	**7.5 (25% ↓)**	105.85	99.70
	200	G-EM	5 (50% ↓)	147.60	114.52
			2.5 (75% ↓)	73.80	57.26
		G-IM	5 (50% ↓)	126.95	104.17
			2.5 (75% ↓)	63.47	52.07
		G-PM	**7.5 (25% ↓)**	121.35	112.17
Fluoxetine	20	G-EM	5 (50% ↓)	140.61	110.35
			2.5 (75% ↓)	70.30	55.18
		G-IM	5 (50% ↓)	129.11	105.87
			2.5 (75% ↓)	64.55	52.94
		G-PM	**5 (50% ↓)**	98.72	88.84
	40	G-EM	**2.5 (75% ↓)**	97.96	73.37
		G-IM		87.66	69.14
		G-PM	**5 (50% ↓)**	124.89	109.68

When co-administered with fluconazole 100 mg, the following S-CIT dose adjustments were necessary to achieve an AUC equivalent to S-CIT monotherapy: 5 mg (reduction by 50%) for G-EM and G-IM and 7.5 mg (reduction by 25%) for G-PM (Figure 6A). At a higher dose of fluconazole 200 mg, incremental S-CIT dose adjustment by 25% did not yield the doses that satisfy the equivalent S-CIT exposure for G-EM and G-IM. As such, the prediction results with two S-CIT doses of 2.5 and 5 mg (reduction by 75% and 50%) are shown (Figure 6A). For co-administration with fluoxetine 20 mg, incremental S-CIT dose adjustment by 25% did not yield the doses that satisfy the equivalent S-CIT exposure for G-EM and G-IM. The prediction results are shown for two S-CIT doses of 2.5 and 5 mg (Figure 6B). On the other hand, an adjusted S-CIT dose of 5 mg (reduction by 50%) led to equivalent S-CIT exposure for G-PM at both doses of fluoxetine (Figure 6B).

## 4 Discussion

The current study quantitatively compared the risk of CYP2C19-mediated DDIs involving S-CIT as a victim drug and four frequently co-prescribed CYP2C19 inhibitors (esomeprazole, omeprazole, fluconazole, and fluoxetine). The real-world patient-level prevalence of concomitant use of the four selected CYP2C19 inhibitors in Korean older patients receiving S-CIT was estimated to be 28.0% (12,278 out of 31,829 patients) (Table 1). Our analysis also indicated that over 10% of Korean older patients received greater than 10 mg S-CIT daily doses together with CYP2C19 inhibitors, exposing older patients to an elevated risk of adverse events from DDI (Figure 1). These results provided an impetus to develop PBPK models that enable quantitative DDI prediction involving S-CIT and CYP2C19 inhibitors, and to develop individualized dosing strategies for S-CIT in older adults. Our current analysis indicated that the extent of DDI involving S-CIT was greater in older adults than in young adults (Figure 3) and varied significantly depending on CYP2C19 phenotypes (Figures 4–6).

Previous PBPK modelling efforts on S-CIT provided important foundations for our current study, in particular, those focused on older adults (Wu et al., 2020; Jang et al., 2025). Expanding on those studies, our current study assessed the combined effect of age and CYP2C19 phenotype on S-CIT PK profiles in older adults. Based on the prediction results with incremental S-CIT dose adjustment, this study proposed the CYP2C19 phenotype-guided dosing strategies in older patients (Table 4). The proposed dosing recommendation may reduce the cases of potentially inappropriate medications and the occurrence of adverse events in older patients.

**TABLE 4:** Summary of recommended S-CIT doses for older adults with differing CYP2C19 phenotypes when co-administered with four CYP2C19 inhibitors.

CYP2C19 inhibitors	Adjusted S-CIT doses for CYP2C19 phenotypes		
	G-EM	G-IM	G-PM
Omeprazole	5 mg	5∼7.5 mg	10 mg
Esomeprazole	5 mg	7.5 mg	10 mg
Fluconazole	5 mg	5 mg	7.5 mg
Fluoxetine	2.5∼5 mg	2.5∼5 mg	5 mg

For guiding S-CIT dose adjustments, we identified reduced doses predicted to yield steady-state AUC values within a bioequivalence range (0.80–1.25) relative to S-CIT monotherapy. This approach was based on the rationale that maintaining equivalent systemic exposure would minimize the risk of subtherapeutic response and concentration-dependent adverse effects. Given that older adults are generally more susceptible to adverse drug reactions and may exhibit heightened PD sensitivity, we adopted a conservative strategy to maintain S-CIT exposures as close as possible to those achieved under monotherapy conditions. This may represent individually optimized conditions that balance efficacy and safety in older patients.

However, for co-administration with fluconazole 200 mg or fluoxetine 20 mg, incremental S-CIT dose adjustment by 25% did not achieve exposures within the targeted bioequivalence range for G-EM and G-IM. Specifically, with fluconazole 200 mg, a 75% dose reduction of S-CIT resulted in AUC values corresponding to 73.80% and 63.47% of S-CIT monotherapy in G-EM and G-IM, respectively (Table 3). Similarly, with fluoxetine 20 mg, a 75% dose reduction yielded AUCs of 70.30% and 64.55% of monotherapy levels in G-EM and G-IM, respectively (Table 3). Although strict bioequivalence to S-CIT monotherapy was not achieved with a 75% dose reduction in these scenarios, the simulated trough plasma concentrations in the majority of populations remained above the minimum therapeutic threshold of 8 ng/mL (Figure 6). Based on this finding, we predict that these reduced doses may still provide adequate therapeutic coverage while mitigating the risk of excessive systemic exposure under DDI conditions.

The current study used the minimum therapeutic boundary of 8 ng/mL (equivalent to 25 nM), supported by previous studies (Gründer et al., 2011; Jukić et al., 2018). The serotonin reuptake inhibition constant for S-CIT was reported to be 9.2 nM (Koldsø et al., 2010). Additionally, Paulzen et al. compared the steady-state concentrations of citalopram between serum and the cerebrospinal fluid (CSF) from 18 patients receiving repeated doses of citalopram (Paulzen et al., 2016). They reported that the citalopram concentrations in the CSF were about one-third of those in the serum. Considering these findings, a minimum therapeutic boundary for S-CIT of 8 ng/mL (25 nM) in plasma was deemed reasonable. However, it remains unclear whether older adults differ from younger adults in CSF drug distribution, serotonin transporter density, or binding sensitivity. As more data on age-dependent changes in target occupancy and the pharmacodynamic effects of S-CIT become available, the minimal therapeutic boundary could be further refined. With respect to the upper boundary, some studies have reported a concentration-dependent increase in the risk of QTc interval prolongation with higher S-CIT levels (Chung et al., 2017). In contrast, others found no direct association between plasma concentrations and QTc interval, instead suggesting that concomitant medications or patient-specific factors play a larger role (Carceller-Sindreu et al., 2017). Given this lack of consensus, we did not define a maximum therapeutic boundary and adjusted S-CIT doses under DDI conditions to ensure bioequivalent S-CIT exposure compared with control conditions.

In older patients, dosing strategies may require careful adjustment due to age-related PK and PD changes, particularly the decline in renal and hepatic function and alterations in drug sensitivity. For instance, renally eliminated drugs such as digoxin, metformin, gabapentin, and direct oral anticoagulants frequently require dose reduction or avoidance when creatinine clearance falls below specific thresholds (O'Mahony et al., 2015; Pazan et al., 2022). Similarly, drugs that undergo extensive hepatic metabolism, including benzodiazepines, opioids, and warfarin, are associated with increased sensitivity in older adults, necessitating lower starting doses and slower titration (“start low, go slow”) (Mangoni and Jackson, 2004). Our current study provides personalized dosing recommendations for S-CIT in older patients, tailored according to CYP2C19 phenotypes and the use of frequently co-administered CYP2C19 inhibitors. Our study has several limitations. First, the PBPK-based dose optimization is a predictive approach that warrants clinical validation. Second, this work did not include PD modelling, and further efforts could integrate PD components to capture potential age-related alterations in serotonin transporter and receptor dynamics (Karrer et al., 2019). Third, implementation of our proposed dose adjustments (Table 4) may face practical and regulatory constraints due to the currently available formulations of S-CIT tablets (commonly 5, 10, and 20 mg) and tablet-splitting concerns (Saran et al., 2022). Fourth, the distribution of CYP2C19 genotypes varies substantially across ethnic groups (Zanger and Schwab, 2013). As our simulations were based on the Geriatric North European Caucasian population, caution is warranted when extrapolating these findings to populations with different metabolizer profiles, such as East Asian cohorts. Fifth, the relatively small size of the validation cohort warrants further investigation to expand the predictive value of our findings.

In conclusion, our current findings underscore the need for a careful risk assessment for DDI when prescribing S-CIT to older patients, especially those receiving four CYP2C19 inhibitors. The phenotype-specific dosing recommendations could serve as a reference for developing personalized therapeutic guidelines to optimize clinical outcomes and minimize adverse effects in this vulnerable population.

## Data Availability

The original contributions presented in the study are included in the article/Supplementary Material, further inquiries can be directed to the corresponding authors.
