# Supplementary material for: Enhanced CYP2C19-mediated drug-drug interaction risk with escitalopram in geriatric populations
Source: Front Pharmacol. 2026 Jan 5;16:1711196. doi: 10.3389/fphar.2025.1711196 (PMC12812941; doi:10.3389/fphar.2025.1711196)
Supplement: Supplementary file 1 [file Supplementaryfile1.pdf]

Supplementary Material

Enhanced CYP2C19-Mediated Drug-Drug Interaction Risk with Escitalopram in Geriatric Populations

Seonyoung Byoun[1], Dong-gyu Heo[2], Minsoo Lee[1], Ryunghwa Lee[1], Yuanyuan Li[1], Ju-Yeon Lee[1], Eunjin Hong[2]*, Wooin Lee[1]*

[1]College of Pharmacy and Research Institute of Pharmaceutical Sciences, Seoul National University, Seoul, Korea

[2]College of Pharmacy, CHA University, Seongnam-si, South Korea

*** Correspondence:**
Eunjin Hong, Ph.D.
ejhong1@cha.ac.kr

Wooin Lee, Ph.D.
wooin.lee@snu.ac.kr

1 Supplementary Results

No results

2 Supplementary Figures and Tables

2.1 Supplementary Figures

Supplementary Figure S1 Comparison of observed and simulated pharmacokinetic (PK) profiles of young and older adults for S-CIT model validation in the control condition. SD values of 168-240 hours in the multiple-dose 10 mg study by Sogaard et al. can't be digitized.

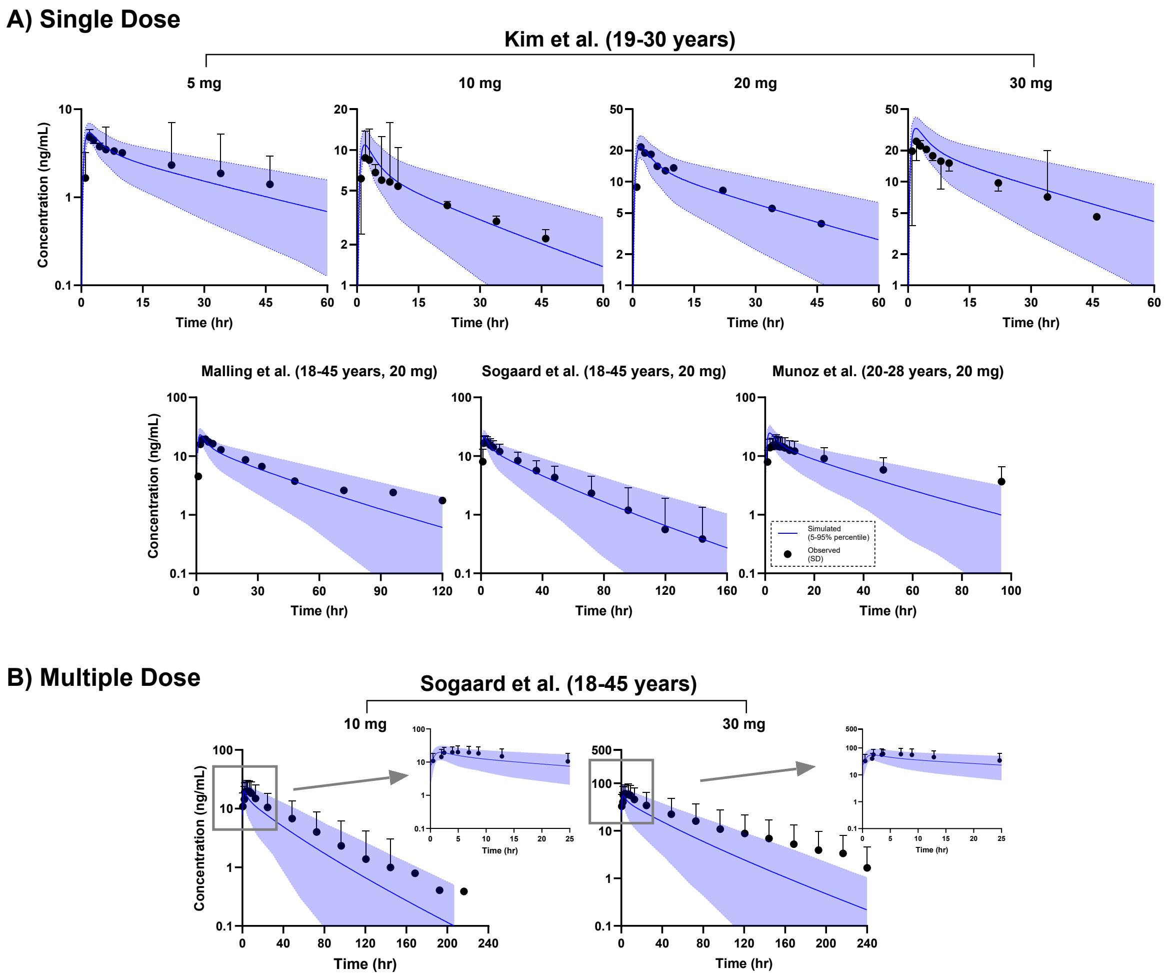

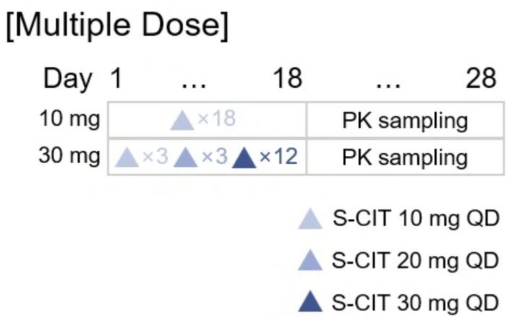

Supplementary Figure S2 Simulated C_{max} and AUC ratios (at steady-state) in non-stratified young and older adults (N=500) for S-CIT under DDI conditions. Distributions are shown as a box-and-whisker plot where the box represents the interquartile range, and the whiskers extend to 10-90%. Geometric means are shown as a dot plot with 90% confidence intervals. (A) omeprazole 40 mg QD, (B) esomeprazole 20 mg QD, (C) fluconazole 200 mg QD, and (D) fluoxetine 40 mg QD. *P* values were determined using a Welch's t-test (assuming unequal variances).

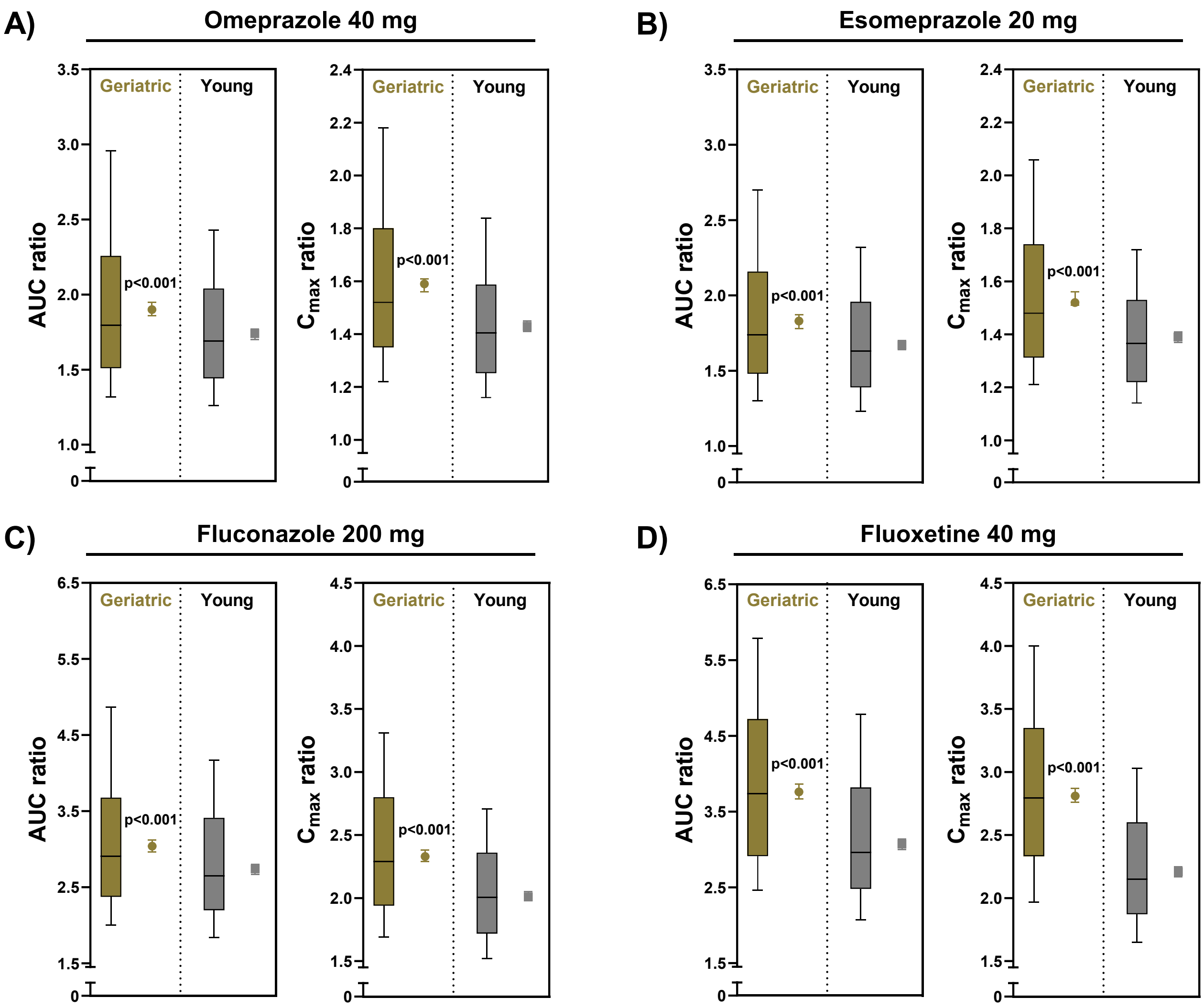

2.2 Supplementary Tables

Supplementary Table S1 Demographic and clinical characteristics of S-CIT users and those with potential DDI (drug-drug interaction) exposure to CYP2C19 inhibitors

	S-CIT users, N (%)	Patients with potential DDI exposure to CYP2C19 inhibitors, N (%)			
		Esomeprazole	Fluconazole	Omeprazole	Fluoxetine
**Patients, N**	**31,829**	**6,179**	**1,138**	**914**	**406**
Age, years, mean ± SD	73.5 ± 8.4	72.8 ± 8.0	72.7 ± 7.7	72.9 ± 7.9	70.1 ± 7.6
60-69	11,361 (35.7)	2,312 (37.4)	432 (38.0)	327 (35.8)	207 (51.0)
70-79	11,679 (36.7)	2,428 (39.3)	455 (40.0)	367 (40.2)	151 (37.2)
80-89	8,218 (25.8)	1,373 (22.2)	243 (21.4)	208 (22.8)	47 (11.6)
90≤	569 (1.8)	66 (1.1)	8 (0.7)	12 (1.3)	1 (0.3)
Sex, Female	21,400 (67.4)	4,308 (69.7)	809 (71.1)	641 (70.1)	272 (67.0)
Depression diagnosis (+)	13,790 (43.3)	2,910 (47.1)	511 (44.9)	416 (45.5)	297 (73.2)

DDI, drug–drug interaction; SD, standard deviation.

Supplementary Table S2 Prevalence of CYP2C19 inhibitor use in Korean geriatric cohorts: A patient-level and person-day analysis in the total population and S-CIT users

**CYP2C19 inhibitors**	**Prevalence in total population (S-CIT users & non-users)** (patient numbers=895,954; person-days=254,762,256)		**Prevalence in S-CIT users** (patient numbers=31,829; person-days=5,531,604)		**Duration of co-administration with S-CIT (days)**	
	Patient-level (Patient numbers, N*)	Person-day (Person-days)	Patient-level (Patient numbers, N*)	Person-day (Person-days)	Mean ± SD	Median (IQR)
Esomeprazole	22.9% (N=205,191)	5.1% (N=13,019,575)	19.9% (N=6,334)	7.4% (N=408,581)	64.5 ± 88.4	27 (9-75)
Cimetidine	20.9% (N=187,573)	1.6% (N=4,097,777)	14.2% (N=4,522)	2.5% (N=140,113)	31.0 ± 59.5	9 (4-26)
Fluconazole	6.1% (N=54,732)	0.4% (N=934,643)	3.8% (N=1,192)	0.4% (N=19,244)	16.1 ± 20.8	10 (5-20)
Lansoprazole	5.9% (N=53,042)	1.6% (N=3,971,619)	6.3% (N=2,001)	2.5% (N=140,671)	70.3 ± 95.9	27 (9-84)
Omeprazole	3.3% (N=29,553)	0.7% (N=1,685,785)	2.9% (N=933)	0.8% (N=46,373)	49.7 ± 73.4	18 (8-61)
Fluoxetine	0.4% (N=3,932)	0.2% (N=541,431)	1.4% (N=459)	0.6% (N=33,823)	73.7 ± 111.4	15 (6-87)
Isoniazid	0.3% (N=2,329)	0.1% (N=260,461)	0.3% (N=84)	0.1% (N=5,293)	63.0 ± 61.1	40 (16.5-92)
Ticlopidine	0.2% (N=1,491)	0.1% (N=370,575)	0.3% (N=109)	0.3% (N=16,934)	155.4 ± 137.1	114 (30-284)
Fluvoxamine	0.1% (N=761)	0.1% (N=134,545)	0.2% (N=72)	0.1% (N=7,094)	98.5 ± 127.5	26.5 (9-146.5)
Voriconazole	0.0% (N=100)	0.0% (N=5,901)	0.0% (N=4)	0.0% (N=58)	14.5 ± 14.8	11 (4-25)
Modafinil	0.0% (N=43)	0.0% (N=6,193)	0.0% (N=9)	0.0% (N=969)	107.7 ± 115.2	42 (28-224)
Armodafinil	0.0% (N=6)	0.0% (N=1,047)	0.0% (N=1)	0.0% (N=120)	120 ± 0	120 (120-120)
**Subtotal**	60.1% (N=538,753)	9.9% (N=25,029,552)	49.3% (N=15,720)	14.7% (N=819,273)	-	-

SD, standard deviation; IQR, interquartile range.

* N represents the total number of patient-dose observations, not unique patients; individuals may be counted in multiple dose categories if their escitalopram dose changed.

Supplementary Table S3 Parameters used for S-CIT PBPK model in Simcyp Simulator (version 23, Certara)

Parameters	Value	Reference
**Physiochemical properties**		
Molecular weight (g/mol)	324.39	Drugbank
Log $P_{o:w}$	3.76	
Compound type	Monoprotic Base	
pKa	9.78	
PSA ($\dot{A}^2$)	36.26	
Hydrogen bond donors	0	PubChem
Hydrogen bond acceptors	4	
B/P	1.351	[1]
fu_p	0.44	LEXAPRO Package insert
**Absorption**		
Absorption model	ADAM	
PAMPA ($\times 10^{-6}$ cm/sec)	14.5	[2]
$P_{eff,man}$ ($\times 10^{-4}$ cm/sec)	2.7652	Predicted
k_a (h^{-1})	2.0498	
f_a	0.99354	
$f_{u,gut}$	10^{-6}	User Input
**Distribution**		
Distribution model	Full PBPK model	
V_{ss} (L/kg)	12.371	Predicted
**Tissue:plasma partition coefficients**		
Adipose	3.26	[3]
Bone	4.8	
Brain	3.32	
Gut	17.42	
Pancreas	10.55	
Heart	18.45	
Kidney	389.16	
Liver	144.52	
Lung	3.29	
Muscle	15.2	
Skin	8.2	
Spleen	17.05	
**Elimination**		
CL_{po} (L/h)	36	LEXAPRO Package insert
CYP2C19 CL_{int} (ml/min/pmol CYP)	2.6795	Predicted in Simcyp using the Retrograde Calculator
CYP2D6 CL_{int} (ml/min/pmol CYP)	0.48834	
CYP3A4 CL_{int} (ml/min/pmol CYP)	0.045243	
CYP2C19 f_m (%)	52	[4]
CYP2D6 f_m (%)	19	
CYP3A4 f_m (%)	29	
Renal clearance (L/h)	2.52	LEXAPRO Package insert

Log $P_{o:w}$, logarithmic partition coefficient octonal:water; pK_a, logarithm of acid dissociation constant; PSA, polar surface area; B/P, blood-to-plasma ratio; fu_p, fraction unbound in plasma; $P_{eff,man}$, effective permeability in man; k_a, absorption rate constant; f_a, fraction available from dosage form; $f_{u,gut}$, fraction unbound in the enterocyte; V_{ss}, volume of distribution at steady state; CL_{po}, in vivo oral clearance; CL_{int}, intrinsic clearance; fm, fraction of drug metabolized.

Supplementary Table S4 Parameters of racemic omeprazole from Wu et al.[5] used for DDI validation

	Racemic omeprazole from Wu et al.			
	R-omeprazole		S-omeprazole	
**Parameters**	**Value**	**Reference**	**Value**	**Reference**
**Physicochemical properties**				
Molecular weight (g/mol)	345.4	[6]	345.4	[6]
Log $P_{o:w}$	2.23		2.23	
Compound type	Ampholyte		Ampholyte	
pKa	4.4, 8.7		4.4, 8.7	
PSA ($\dot{A}^2$)	77.1	Simcyp v23	77.1	Simcyp v23
Hydrogen bond donors	1		1	
Hydrogen bond acceptors	6		6	
B/P	0.59	[6]	0.59	[6]
fu_p	0.04		0.03	LEXAPRO Package insert
**Absorption**				
Absorption model	First-Order		First-Order	
MDCK 2 ($\times 10^{-6}$ cm/sec)	No need for the First-Order option.			
$P_{eff,man}$ ($\times 10^{-4}$ cm/sec)				
k_a (h^{-1})	2	Estimated by NCA	10	Estimated by NCA
f_a	0.99645	Simcyp v23	0.99645	Simcyp v23
$f_{u,gut}$	0.04	Assumed same as fu_p.	0.03	Assumed same as fu_p.
Q_{gut} (L/h)	11.4	Predicted	6	Predicted
**Distribution**				
Distribution model	Minimal PBPK model		Minimal PBPK model	
V_{ss} (L/kg)	0.11	Sensitivity analysis	0.2	[7]
**Elimination**				
CYP2C19 CL_{int} (ml/min/pmol CYP)	29.9	Predicted in Simcyp using the Retrograde Calculator	24.3	Predicted in Simcyp using the Retrograde Calculator ([7])
CYP3A4 CL_{int} (ml/min/pmol CYP)	0.0624		0.36	
CYP2C19 fm (%)	98	[8]	73	[8]
CYP3A4 fm (%)	2		27	
Renal clearance (L/h)	0.037	[6]	0.037	[6]
**Interaction**				
**Competitive inhibition**				
CYP2C19 K_i (uM)	4.05	[6] (Assume Ki = IC50/2, IC50 = 8.1 uM)	7.5	[6] (Assume Ki = IC50/2, IC50 = 8.1 uM)
CYP2C19 $f_{u,mic}$	1	Simcyp compound library	1	Simcyp compound library
**Mechanism-based inhibition**				
CYP2C19 K_{app} (uM)	1.6	Sensitivity analysis	0.3	Sensitivity analysis
CYP2C19 k_{inact} (h^{-1})	4		5	
CYP2C19 $f_{u,mic}$	1	Simcyp v23	1	Simcyp v23

Log $P_{o:w}$, logarithmic partition coefficient octonal:water; pK_a, logarithm of acid dissociation constant; PSA, polar surface area; B/P, blood-to-plasma ratio; fu_p, fraction unbound in plasma; $P_{eff,man}$, effective permeability in man; k_a, absorption rate constant; f_a, fraction available from dosage form; $f_{u,gut}$, fraction unbound in the enterocyte; $Q_{gut,}$ flow rate for overall delivery of drug to the gut; V_{ss}, volume of distribution at steady state; CL_{po}, in vivo oral clearance; CL_{int}, intrinsic clearance; fm, fraction of drug metabolized.

Supplementary Table S5 Comparison of observed and simulated pharmacokinetic parameters of young and geriatric adults for S-CIT model validation in the control condition

Study details				PK parameters (Mean (SD))				Ratio	
				Observed		Simulated		Simulated /Observed	
**Regimen**	**Ref**	**Age**	**Dose (mg)**	**C_{max} (ng/mL)**	**AUC (h·ng /mL)**	**C_{max} (ng/mL)**	**AUC (h·ng /mL)**	**C_{max}**	**AUC**
Single dose	[9]	19-30	5	4.81 (0.82)	195.68 (79.39)	5.53 (0.82)	140.94 (66.05)	1.15	0.72
			10	8.74 (0.98)	357.21 (185.18)	11.06 (1.65)	281.89 (132.10)	1.27	0.79
			20	21.59	530.30	22.12 (3.29)	563.77 (264.19)	1.02	1.06
			30	24.50 (1.53)	673.61 (42.00)	33.17 (4.94)	845.66 (396.29)	1.35	1.26
	[10]	18-45	20	20.10	600.30	23.50 (4.40)	569.00 (262.80)	1.17	0.95
	[11]	18-45		18.80 (4.50)	637.70 (356.20)	22.10 (3.30)	560.40 (261.50)	1.17	0.88
	[12]	20-28		15.70 (7.30)	740.90 (354.00)	25.20 (4.80)	536.60 (218.50)	1.60	0.72
Multiple dose (steady-state)	[13]	18-35	10	22.70 (7.50)	362.20 (146.00)	19.80 (6.00)	287.00 (132.30)	0.87	0.79
		65-80		30.50 (12.50)	544.30 (218.60)	31.60 (12.20)	530.70 (273.50)	1.04	0.97
	[11]	18-45		20.60 (10.30)	360.10 (218.80)	19.90 (6.00)	289.30 (132.60)	0.97	0.80
			30	64.40 (33.70)	1101.00 (733.80)	55.50 (17.40)	840.50 (392.10)	0.86	0.76

PK, pharmacokinetic; Cmax, maximum plasma concentration; AUC, area under the curve (AUC (0-∞) for single dose studies except for Munoz et al., AUCτ for Munoz et al., and multiple dose studies); SD, standard deviation.

In Kim et al., the doses of 5, 10, 20, and 30 mg had sample sizes of N = 4, 4, 1, and 3, respectively. For the 20 mg group (N = 1), the standard deviation could not be calculated.

Supplement Table S6 Comparison of observed and simulated C_{max} and AUC ratio (at steady-state) for DDI validation of omeprazole (CYP2C19 inhibitor) with clopidogrel (CYP2C19 substrate) [14]

PK parameters	Day	Condition	Observed (SD)	Simulated (SD)	Ratio (Simulated/Observed)
Cmax (pg/mL)	Day 1	Control	5,240 (6,290)	10,460 (27,310)	2.00
		DDI	5,560 (7,920)	11,220 (28,860)	2.02
		Ratio (DDI/control)	**1.06**	**1.07**	**1.01**
	Day 5	Control	1,770 (3,370)	1,960 (3,410)	1.11
		DDI	1,980 (2,950)	2,290 (4,280)	1.16
		Ratio (DDI/control)	**1.12**	**1.17**	**1.04**
AUC (h·pg/mL)	Day 1	Control	10,400 (10,500)	19,240 (28,050)	1.85
		DDI	13,600 (15,500)	24,580 (37,380)	1.81
		Ratio (DDI/control)	**1.31**	**1.28**	**0.98**
	Day 5	Control	2,880 (4,230)	4,000 (4,530)	1.39
		DDI	4,000 (4,580)	5,110 (6,400)	1.28
		Ratio (DDI/control)	**1.39**	**1.28**	**0.92**

CYP2C19 substrate: Clopidogrel (Day 1: 300 mg QD, Day 2~5: 75 mg QD)
CYP2C19 inhibitor: Omeprazole (80 mg QD, 5 days)
Day 1: pre-clopidogrel loading dose, Day 5: steady-state clopidogrel

1. Fisar, Z., et al., *Distribution of antidepressants between plasma and red blood cells.* Neuroendocrinol. Lett., 2006. **27**(3): p. 307-313.
2. Feng, B., et al., *In Vitro P-glycoprotein Assays to Predict the in Vivo Interactions of P-glycoprotein with Drugs in the Central Nervous System.* Drug Metab Dispos, 2008. **36**(2): p. 268-275.
3. Wu, X., et al., *Physiologically Based Pharmacokinetic Approach Can Successfully Predict Pharmacokinetics of Citalopram in Different Patient Populations.* J. Clin. Pharmacol., 2020. **60**(4): p. 477-488.
4. Zhou, L., et al., *Assessing pharmacokinetic differences in Caucasian and East Asian (Japanese, Chinese and Korean) populations driven by CYP2C19 polymorphism using physiologically-based pharmacokinetic modelling.* Eur. J. Clin. Pharmacol., 2019. **139**: p. 105061.
5. Wu, F., et al., *Predicting Nonlinear Pharmacokinetics of Omeprazole Enantiomers and Racemic Drug Using Physiologically Based Pharmacokinetic Modeling and Simulation: Application to Predict Drug/Genetic Interactions.* Pharm Res, 2014. **31**(8): p. 1919-1929.
6. Ogilvie, B.W., et al., *The Proton Pump Inhibitor, Omeprazole, but Not Lansoprazole or Pantoprazole, Is a Metabolism Dependent Inhibitor of CYP2C19: Implications for Coadministration with Clopidogrel.* Drug Metab Dispos, 2011. **39**(11): p. 2020-2033.
7. Hassan-Alin, M., et al., *Pharmacokinetics of esomeprazole after oral and intravenous administration of single and repeated doses to healthy subjects.* Eur. J. Clin. Pharmacol., 2000. **56**(9-10): p. 665-670.
8. Andersson, T. and L. Weidolf, *Stereoselective disposition of proton pump inhibitors.* Clin Drug Investig, 2008. **28**(5): p. 263-279.
9. Kim, E., et al., *Regional Differences in Serotonin Transporter Occupancy by Escitalopram: An [(11)C]DASB PK-PD Study.* Clin Pharmacokinet, 2017. **56**(4): p. 371-381.
10. Malling, D., M.N. Poulsen, and B. Sogaard, *The effect of cimetidine or omeprazole on the pharmacokinetics of escitalopram in healthy subjects.* Br J Clin Pharmacol, 2005. **60**(3): p. 287-290.
11. Sogaard, B., et al., *The Pharmacokinetics of Escitalopram After Oral and Intravenous Administration of Single and Multiple Doses to Healthy Subjects.* J. Clin. Pharmacol., 2005. **45**(12): p. 1400-1406.
12. Munoz, E.O., D.; Yepes, N., *Bioequivalence Study of Two Formulations of Escitalopram Oxalate 20 mg Tablets in Healthy Volunteers.* J Bioequiv Availab, 2015. **7**(5): p. 205-209.

13. Gutierrez, M. and H. Mengel, *Pharmacokinetics of escitalopram*, in *National Institute of Mental Health / 42nd Annual New Clinical Drug Evaluation Unit Meeting*. 2002: Boca Raton, FL.

14. Angiolillo, D.J., et al., *Differential Effects of Omeprazole and Pantoprazole on the Pharmacodynamics and Pharmacokinetics of Clopidogrel in Healthy Subjects: Randomized, Placebo-Controlled, Crossover Comparison Studies.* Clin. Pharmacol. Ther., 2011. **89**(1): p. 65-74.
